# Cancer and Mediterranean Diet: A Review

**DOI:** 10.3390/nu11092059

**Published:** 2019-09-02

**Authors:** Maria Chiara Mentella, Franco Scaldaferri, Caterina Ricci, Antonio Gasbarrini, Giacinto Abele Donato Miggiano

**Affiliations:** 1 UOC di Nutrizione Clinica, Area Medicina Interna, Gastroenterologia e Oncologia Medica, Dipartimento di Scienze Gastroenterologiche, Endocrino-Metaboliche e Nefro-Urologiche, Fondazione Policlinico Universitario A. Gemelli IRCCS, Università Cattolica del Sacro Cuore, 00168 Rome, Italy; 2 UOC di Medicina Interna e Gastroenterologia, Area Medicina Interna, Gastroenterologia e Oncologia Medica, Dipartimento di Scienze Gastroenterologiche, Endocrino-Metaboliche e Nefro-Urologiche, Fondazione Policlinico Universitario A. Gemelli IRCCS, Università Cattolica del Sacro Cuore, 00168 Rome, Italy; 3 UOC di Ginecologia Oncologica, Area Salute della Donna, Dipartimento di Scienze della Salute della Donna, del Bambino e di Sanità Pubblica, Fondazione Policlinico Universitario A. Gemelli IRCCS, Università Cattolica del Sacro Cuore, 00168 Rome, Italy

**Keywords:** Mediterranean diet, cancer incidence, cancer

## Abstract

The Mediterranean diet is considered one of the most worldwide healthy dietary patterns thanks to a combination of foods rich mainly in antioxidants and anti-inflammatory nutrients. Many studies have demonstrated a strong and inverse relationship between a high level of Mediterranean diet adherence and some chronic diseases (such as cardiovascular diseases, diabetes, etc.) and cancer. Given its protective effects in reducing oxidative and inflammatory processes of cells and avoiding DNA damages, cell proliferation, and their survival, angiogenesis, inflammations and metastasis, the Mediterranean diet is considered a powerful and manageable method to fight cancer incidence. The aim of this narrative review was to determine the magnitude of interaction between the Mediterranean diet and more widespread types of cancer so as to give a first and useful overview on this relationship identifying, with a nutritional approach, those nutrients of Mediterranean diet able to reduce cancer incidence.

## 1. Introduction

Cancer is the second leading cause of death globally: In 2018 alone, it was registered that about 9.6 million deaths were because of cancer in the ratio of one to six [1]. According to the World Cancer Research Fund and with reference to 2018, 18 million of new cases were diagnosed: Globally, a higher incidence of lung and breast cancer (incidence of 12.3% respectively) was registered, followed by a high incidence of colorectal cancer (10.6%), prostate cancer (7.5%), stomach cancer (6.1%) and liver cancer (5.0%) [2]. Although cancer develops with an homogeneous distribution between men (9.5 million of new diagnoses in 2018) and women (8.5 million of new cases in 2018), each type of neoplasia has a different incidence among male and female: In 2018, lung cancer (15.5%), prostate cancer (14.4%), and colorectal cancer (11.4%) affected men above all, while women have been mainly affected by breast cancer (25.4%), colorectal cancer (9.7%) and lung cancer (8.8%) [2].

Risk factors in cancer incidence are mainly linked with individual and environmental characteristics (heredity, particular exposure to noxious substances, or clinical conditions as hormone imbalance) or with peoples’ lifestyle: Physical activity, sedentary lifestyle and diet play a crucial role. Particularly, a high body mass index, a low intake of fruit and vegetables, the lack of physical activity, the use of alcohol and smoking represent the five and most important risk factors in the onset of several neoplasia, together with some chronic infections (e.g., due to Helicobacter pylori, Human papillomavirus, Hepatitis B, Hepatitis C and Epstein-Barr virus) [3,4,5,6] and with a lack of prevention, screening and treatment of cancer, typical shortages of developing countries especially [1].

Although smoking is the main factor risk, considered as the leading cause of 22% cancer death, the other factors, together with genetic and hereditary characteristics of each individual, contribute decidedly towards the transformation of healthy cells into cancer cells through a progression, more or less quickly able to change precancerous lesions in malignancies [7,8,9].

Diet, physical inactivity, sedentary lifestyle, and obesity (consequence of a healthy or non-healthy lifestyle) are, after smoking, the main risk factors in the onset of cancer: It is estimated that changes in alimentary habits can contribute to avoid cancer onset of 30–50% [10,11].

Dietary patterns based on regular intake of fruit, vegetables (especially garlic and cruciferous vegetables, as cabbages, broccoli, brussels sprout and wasabi) and by consequence the intake of aliments rich in selenium, folic acid, vitamins (B-12 or D), and antioxidants (e.g., carotenoids and lycopene) play a protective role in cancer onset so to reduce risk of breast cancer, colorectal cancer and prostate cancer of 60–70% and of lung cancer; 40–50% [10]. A high intake of products rich in fiber (e.g., whole grains) and a moderate intake of milk and dairy may reduce incidence of different types of cancer (e.g., colorectal cancer, lung cancer, stomach cancer, breast cancer, colorectal cancer, esophagus cancer and oral cancer) [2,12,13,14,15,16,17]. Vice versa, meat and animal products, rich in animal fats and oils and often cooked at high temperatures, may increase cancer incidence, especially for colorectal cancer, stomach cancer and prostate cancer [13,18,19,20,21,22,23].

In regards to alcohol intake and its positive or negative effects on health, there is no unanimity among studies: A moderate alcohol intake (until 30 g a day) could have a protective effect against the onset of kidney cancer, while an excessive intake surely is a risk factor in the onset of many cancers (oral cavity cancer, esophagus cancer, breast cancer, colorectal cancer, stomach cancer and liver cancer) [2].

Summarizing, from several studies emerged as the regular assumption of fruits and vegetables rich in fiber and vitamins, a low intake of meat, and a moderate intake of milk, dairy, and alcohol may be considered an optimal combination in the prevention of cancer. Combining these evidences with the healthier dietary patterns proposed as emerging medical prescription [24], the Mediterranean diet (MD) appears as the best diet pattern able to reflect many characteristics of an ideal healthy diet. It is considered one of the main dietary patterns able to give beneficial effects on longevity and to improve cardiovascular system functions so as to prevent many cardiac diseases or to contain their progression [25,26].

Given these assumptions, the main aim of this narrative review was to understand as Mediterranean diet may contribute to reduce cancer incidence analyzing the impact of MD on more kinds of neoplasia so to understand which areas of nutrition should be deepened to ensure better nutrition guidelines for global population and better rules of thumb in oncologic clinical practice. The novelty of this review is given by the evaluation of the MD impact only considered those studies which demonstrate a significant interaction between MD adherence and cancer. This choice was supported by two considerations. The first is inherent to the statistical results: The lack of significance means a lack of evidence which could be determined by a lack of the actual relationship or by a non-correct approach to the problem. For MD, this last issue is more evident given the complexity of the MD components due to the variety of foods. As emerged in some studies, there are two kinds of problems producing MD complexity: Several myths and misconceptions are associated with the traditional Mediterranean diet, and many difficulties are related with a correct distinction between Mediterranean foods and healthy, but non-Mediterranean foods [27]. The second reason of our work is related to the high heterogeneity [28] of results which does not allow to define a unique and determined relationship between MD and cancer.

## 2. Methodology

Through the analysis of several studies of the last 10 years (2009–2019) about the relationship between the Mediterranean diet and cancer incidence, excluding studies which, although methodologically correct, had not reported significant relationships, we marked out principal scientific facts with specific attention to the most widespread neoplasia, underlining for each of them the significant relationship and the main benefits of Mediterranean diet in the reduction of cellular mutation and in the slowdown of progression and spread of that determined pathology. This study started with a research on PubMed of a series of keywords related to Mediterranean diet and its interaction with some prevalent neoplasia (“Mediterranean diet”, “Mediterranean adherence”, “cancer”, “lung cancer”, “breast cancer”, “colorectal cancer”, “prostate cancer”, “bladder cancer”, “stomach cancer”, “endometrial cancer”, “cervical cancer”, “pancreatic cancer”, “bile tract cancer”). Types of cancer were selected in accordance with their percentage of incidence among worldwide population.

**Figure 1:**
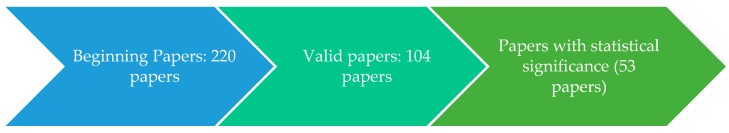
Article selection process. Note: Papers with significant relationship were reported in bibliography together with the other articles considered to describe the whole topic.

Afterwards, we considered only prospective or retrospective studies, excluding systematic reviews and meta-analyses. The last update of our dataset was performed in June 2019. Initially, we examined 220 articles; after the selection process (Figure 1), we identified 104 papers whose 53 characterized by a significant relationship between MD and cancer.

## 3. Mediterranean Diet

The Mediterranean diet was recognized as “intangible cultural heritage of France and Italy, Greece, Spain and Morocco, respectively” by the United Nations Educational, Scientific and Cultural Organization (UNESCO) in 2010 [29], thereby to preserve the local biodiversity characteristics of Mediterranean countries and to promote a diet pattern able to give beneficial effects to human health. Considered for the first time by Ancel Keys and his colleagues as a diet poor in saturated lipids and able to protect cardiovascular system thanks to low cholesterol level in the blood [30], in the following years it was identified as a diet pattern composed by foods rich in high protective nutrients able to prevent from several diseases. Besides, given the protective role plays by MD on DNA damages, it was often investigated in the evaluation of general mortality as a potential and beneficial lifestyle in the increasing longevity in healthy people [30,31].

Mediterranean diet is characterized by a high intake of vegetables, legumes, fresh fruit, non-refined cereals, nuts, and olive oil (especially extra-virgin olive oil, i.e., obtained with a mechanical pressing and contained acidity rate lower than 0.8% (Regulations CEE n. 2568/91 and following updating [32]), by a moderate consumption of fish and dairy, by a low intake of red meats, and by a moderate use of ethanol, mainly red wine consumed during the main meals [26].

In Mediterranean countries, these foods are daily consumed, following precise and traditional habits: Olive oil is taken every day and jointly to main dishes (vegetables and legumes) so to improve their taste, fresh fruit is a form of dessert consumed at the end of the main meals or as a snack for mid-morning or in the afternoon, cheese is consumed with salad or stews and finally, red meat is the main dish of special occasion only (until 1970 in some areas of the Southern Italy or in Greece, meat intake consisted of annual consumption of one/two tame ungulate animals for each patriarchal family and of a moderate intake of poultry) [33,34,35,36].

**Figure 2:**
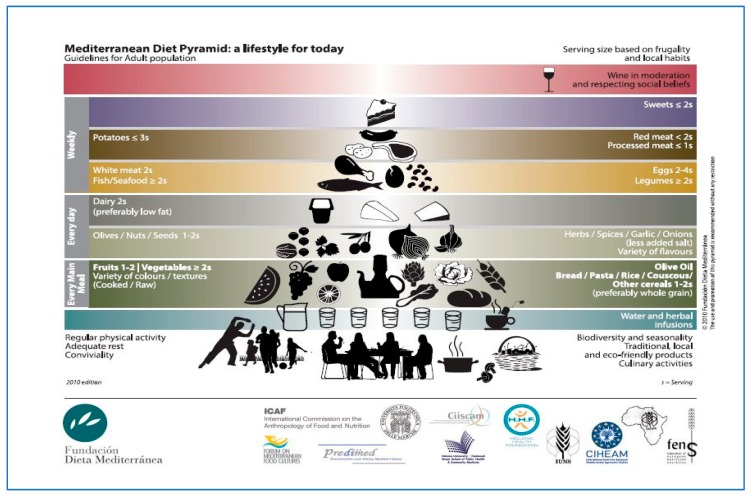
Mediterranean diet pyramid. Source: Fundacion Dieta Mediterrànea.

Given similar climatic and morphological features, distinctive traits of the whole Mediterranean area, the “classical” Mediterranean diet, as described above, has some specific peculiarities depending on the specificities of each country and even on the particularity within a certain area [36,37]. Therefore, for studying Mediterranean diet and its benefits on health, it has been necessary to identify common characteristics among different models of Mediterranean diet, so to creating an alimentary pyramid (Figure 2) in which main foods of each specific kind of Mediterranean diet and their intake frequency were reported, so to uniform different Mediterranean diet models with reference to both foods and their right intake quantity.

Observing Figure 2, it is possible to note as the diet in its largest meaning, i.e., comprehensive of all specificities related to each Mediterranean area, is a diet pattern which promotes a high intake of wholegrain cereals, fruit and vegetable, a moderate consumption of dairy, poultry and fish and a low intake of red meat and sweets. Wine consumption is allowed, on condition that its use is moderate, while olive oil is recommended as fats substitute in each main meal [38]. Regular physical activity, adequate rest, conviviality, biodiversity and seasonality, traditional, local and eco-friendly products and culinary activities are an integral part of Mediterranean diet pyramid.

Mediterranean diet is often compared with some other healthy dietary patterns, as the Nordic diet, dietary approaches to stop hypertension (DASH), the Alternate Health Index (AHEI)-2010 or the vegetarian diet. All these types of diets have two kinds of characteristics: They are derived from precise guidelines and they are formed including or excluding specific foods. For example, the Nordic diet, one of the most studied healthy diet, is composed with healthy foods, as whole-grain cereals, fish, cabbage, rye bread, apples and pears, and root vegetables, but it is also characterized by unhealthy foods as margarine and sugar [28,39], so its impact on health is always evaluated with attention to a single food and not as an alimentary habits on the whole. The same considerations are possible about vegetarian diet: In this case, the choice to eliminate fish, meat and poultry consumption if on the one hand it improves body mass index, reducing obesity and overweight incidences, and reduces the amounts of saturated fatty acids, on the other hand it decreases levels of vitamin B12 and sex hormones, and it increases levels of homocysteine [40]. In both cases, the improvement in the disease’s prevention is linked with diet capacity to contrast or prevent some risk factors, as obesity, overweight or hormonal changes, but they do not provide a 360-degree protective and healthy effect.

On the contrary, the Mediterranean diet presents many advantages related with individual characteristics and exogenous values related with the preservation of traditions and habits and with the environmental protection [41]. The Mediterranean diet is characterized by positive, healthy and nutritional benefits as emerged by the large literature dedicated from 1960 to this dietary pattern; particularly the added value is due to a combination of healthy foods and their many nutritional benefits (high intake of vitamins and nutrients) with the diversity of foods (from vegetables and fruits to meat and fish, without alcohol ban), the respect of seasonality of each ingredient, the freshness of products so to maximize the content of protective nutrients and substances, and the use of all available foods, sweets included, in a context of frugality and moderation [42,43,44].

Since MD is not just a way of eating specific food, but it is a kind of philosophy involving all dimensions of an individual, the Mediterranean diet impacts on life habits changing individual approach to food and the use itself of food [44]. In fact, adhering to MD means learning a variety of food practices, food preparations skills, and culinary activities combining the pleasant tastes and smells of foods with the Mediterranean knowledge and traditions transmitted from generation to generation, living the meal time as a fundamental part of daily routine in which sociability, communication and conviviality engage a psychophysical dimension [43].

**Figure 3:**
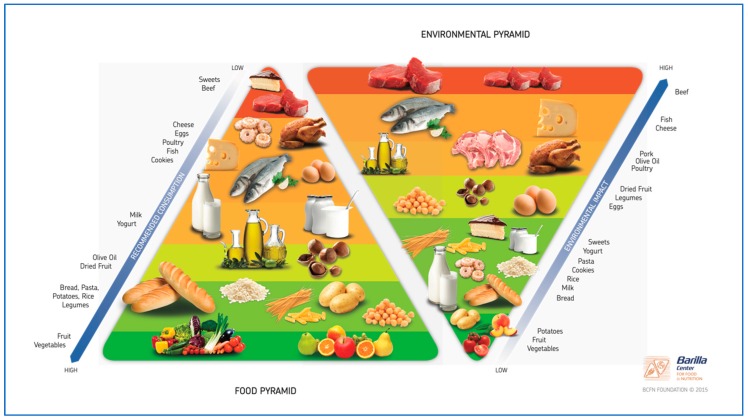
Double Pyramid proposed by Barilla Centre for Food and Nutrition—Source: Barilla Center For Food and Nutrition (https://www.barillacfn.com/en/dissemination/double_pyramid/).

In terms of socio-economic and environmental factors, the choice to adopt a Mediterranean diet means to reduce the impact of food production on environment, reducing food processing loss, food wastage and packaging, energy consumption, transportation of foods, water consumption, and waste management, all environmental issues increased by a strong demand of meat and dairy products and of products no-linked with local and traditional habits. It derives that relationship between nutrition and environmental is inversely associated: More healthy, lower environmental impact [45], as emerged in the Double Pyramid proposed by Barilla Centre for Food and Nutrition in June 2009 (Figure 3). Environmental protection, although in the short run it does not seem linked with the onset of many disease, has a powerful effect in the long period: Factory farming or intensive cultivations have an enormous impact on environmental increasing greenhouse gas emission, exploitation of land and water sources, and pollution, among other reasons [42].

### 3.1. Mediterranean Diet: Open Issues

In accordance with definition of UNESCO, the Mediterranean diet is not a mere collection of some specific foods, but it is a way to promote social interaction and to increase sociocultural values thanks to the sharing of socialization moments and the respect for the territory and biodiversity, ensuring at the same time, the conservation and development of all those traditional activities typical of the Mediterranean basin, and the promotion of environmental conservation [29].

Nevertheless, increasing the adherence to MD is difficult because there are two fundamental aspects to taking in account: One is concerned with the economic accessibility of Mediterranean foods for all people [24,42,46] (it is estimated that world’s population will reach to nine billion people by 2050 [47]), and the second is linked with the transportation of Mediterranean foods in non-Mediterranean countries.

With attention to the first issue, the reasons to a low level of adherence are mainly linked with socio-economic factors and with an increase of prices of major food items involved in the MD pyramid. More specifically, people, especially disadvantaged people, are induced to choose less expensive eating patterns or to prefer energy-dense foods, i.e., those composed by refined grains, added sugar, and fats, because these foods are the lowest-cost option and the best source of energy [48]. Subjects reporting highest scores for the Western diet habits demonstrate (consciously or unconsciously) to protect the economic aspect rather than to enhance their diet quality, even if this behavior affects their health status (higher rates of obesity, diabetes, cardiovascular diseases, and some type of cancer are more common among lower socio-economical group than among better educated and more affluent people) [46,49,50].

The second issue is more complicated to solve. In fact, while socio-economic problems could be tackled with a major attention to public and global politics lowering the prices of some basic products as fruits and vegetables and improving their availability in the most citizen-attended places, the second issue regards a choice between sustainability and health. In fact, while reintroducing MD is easy in Mediterranean countries because it requires a change of individuals’ economic status, its transferability in non-Mediterranean countries, with all its aspects, traditional components included, is not a simply shifting of foods from an area to another, but it is a change in dietary habits [27].

### 3.2. Scores for Measuring the MD Adherence

Since the Mediterranean diet is given by a combination of several foods, it is impossible to determine which specific food produces a positive effect and contributes to fight certain pathologies. Therefore, over the years, it has been necessary to define new research methodologies (general description, dietetic pyramids, a priori or a posteriori scoring systems [51]) able to evaluate the whole diet pattern, tools that, although different in their estimate methods, aim to understand the level of adherence to MD of each individual involved in a specific study. Particularly, a priori and a posteriori scoring systems (a priori: Default score determined using nutritional data of each subject, e.g., the Mediterranean Diet Score (MDS) or the Italian Mediterranean Index; a posteriori: Score determined after a principal component analysis which defines diet pattern, e.g., alternate Mediterranean Diet (aMED) or Alternate Health Index (AHEI)) [52], used in many observational and prospective studies, aimed to determine, through an assessment of a MD score per individual, the incidence of each disease and the overall and disease-specific mortality rate. Thanks to these methodologies, they could state with absolute certainty that there is an inverse and significant correlation between MD and overall mortality rate (by 20–30%) and between MD and cardiovascular mortality (by 30–40%) [25,53,54,55,56,57].

All these methodologies, although they are statistically valid, do not consider that characteristic which, for who writes, is the main feature of the MD, i.e., that factor which differentiates this diet pattern from the whole kinds of healthy diets: Naturality. In fact, MD was not developed using healthy guidelines or recommendations, but it is part of the alimentary regimen of Mediterranean countries since Egyptian Pharaohs, and it was proposed as a healthy diet pattern in 1634 [52], becoming integral part of heritage of many generations. Therefore, MD is not a regimen scientifically imposed or a diet in its negative meaning, but it is a normal and traditional alimentary behavior which, jointed with a healthy lifestyle (e.g., physical activity and exposure to sunlight) may effectively reduce the onset of many diseases, without upsetting the individual’s approach to food, increasing indeed the sense of fulfilment and satisfaction.

In fact, although the MD development in the Southern Europe suffered in the last 20–25 years, a strong reduction because of a gradually abandon of Mediterranean alimentary traditions for alimentary habits rich in saturated fats and pre-packaged foods [58,59,60,61,62], the presence in those areas of typical Mediterranean products (as olive oil, tomatoes, vegetables, legumes, and dairy) continues to produce positive effects in the reduction of tumoral incidence in the population [63,64].

### 3.3. Beneficial Effects of Mediterranean Diet in the Cancer Prevention

The positive relationship (beneficial effects) between the Mediterranean diet and cancer is due to the high contents of antioxidants and anti-inflammatory nutrients contained in many foods of MD (legumes, fresh fruit or nuts, vegetables, fish, and olive oil, especially extra-virgin olive oil), which have a protective effect in the fighting cell degeneration and proliferation of cancer cells [65].

Paying attention to the relationship of specific foods and cancer, protective effects of the Mediterranean diet may be assigned to the high polyphenols concentration contained in olive oil, wine, and vegetables, all foods known for their antioxidant and anti-inflammatory capacity [66,67,68,69], and are rich in nutrients able to reduce proliferation of cancer cells, and to protect cell membrane from metastasis [67].

Besides, fruit and vegetables have a high quantity of carotenoids and vitamins, as vitamin C and E, folates and flavonoids, nutrients known for their antioxidant properties which allow the prevention of DNA damages [70,71]. Finally, omega-3, contained abundantly in fish, especially in sardines and mackerel (typical products of Mediterranean diet), and in nuts (almonds, walnuts, and pumpkin seeds) help to slow down cancer development affecting cell proliferation, their survival, angiogenesis, inflammations and metastasis [72]. A low consumption of meat contributes to moderate noxious effects due to high-temperature meat cooking, as well as to reduce animal fats intake [73].

Several studies demonstrated that a high adherence to MD is often associated with a lower risk of malignancies [7,9,15,69,74,75,76,77,78,79,80], although other studies showed a difficulty to identify a direct relationship between MD and cancer because of confounding factors or unclear definition of the MD score. The reason of these difficulties may be identified in the fact that it is impossible to establish a connection between MD adherence and cancer onset, this being determined by a cell degeneration produced by the influence of several factors; this impossibility is therefore due to a lack of a linear relationship between the two variables (MD adherence and cancer) [81] and to a presence of several variables, sometimes not directly perceptible, which modifies (increasing or reducing) the strength of the same relationship [82,83,84] or eliminates its linearity [85].

**Figure 4:**
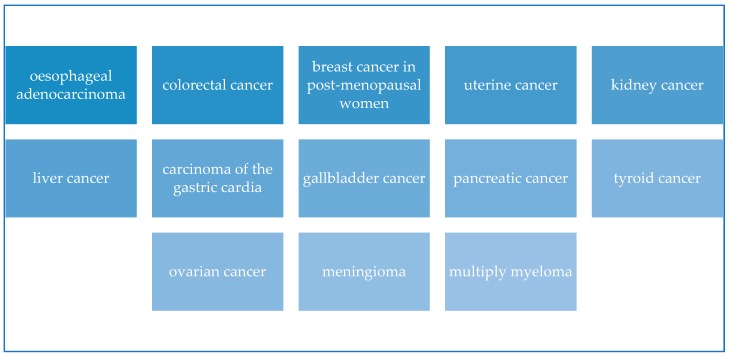
Cancers linked with obesity or overweight—Source information: Lauby-Secretan et al. (2016) [86].

Diet and nutrition are two lifestyle factors having repercussion on the incidence of cancer, but the lack of significance of some studies and the lack of a direct relationship between nutrition and cancer reveal that other lifestyle factors (identified as confounding factors as physical inactivity and sedentary lifestyle) may contribute to the increasing cancer incidence worldwide. Managing properly diet, nutrition, and physical activity and reducing sedentary lifestyles could prevent more than half of cancers occurring today (Kerr 2017) thank to their contribution to reduce obesity which is a known risk factor for many malignancies (being overweight or obese increases the risk of incidence of at least 13 types of cancer (Figure 4)) [86]. Nevertheless, we underline that the Mediterranean diet is considered a form of lifestyle (as explicitly reported in Diet Pyramid in Figure 2) in which food and daily activities participate to realize the original Mediterranean diet. Therefore, we believe that the present methodology that provides for a clear separation between the Mediterranean diet and physical activity is not entirely correct. In fact, we imagine that this approach could be exactly the cause of the lack of confirmation regarding the association between physical activity, Mediterranean diet and cancer risk. In fact, the variety of neoplasia, etiologic differences among them, and the different impact of risk factors cause the lack of unanimity among researches because it was sometimes impossible to determine the cancer risk correlated directly with Mediterranean diet. Some studies demonstrated uniformly that there is a difference cancer incidence between Mediterranean and non-Mediterranean countries supposing that this difference is the result of the combination of many factors linked with diet, physical activity, quality of life and a higher exposure to sunlight [52]. But many biological questions could be behind this association. For this reason, in the following paragraph we provided a short explanation of the principal mechanisms about the relationship between Mediterranean foods and types of cancer.

### 3.4. Mechanisms between Mediterranean Diet Foods and Types of Cancers

**Figure 5:**
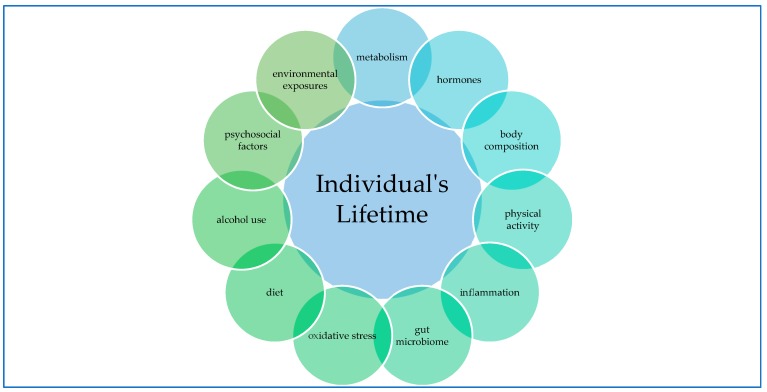
Exposome—Characteristics extracted by Mayne et al. (2016).

The biology of cancer is known to be heterogeneous and this variability is one of the issues which contributes to alter the relationship between dietary factors and cancer risk [87]. Diet is a part of an exposome that encompasses all of the exposure experienced by an individual over his or her lifetime (Figure 5). This means that mechanism involved in the cancer are so dynamic and intertwined that analyzing their effect is complex and that a cause–effect relationship between diet and cancer should be evaluated in the context of the individual’s exposome.

**Figure 6:**
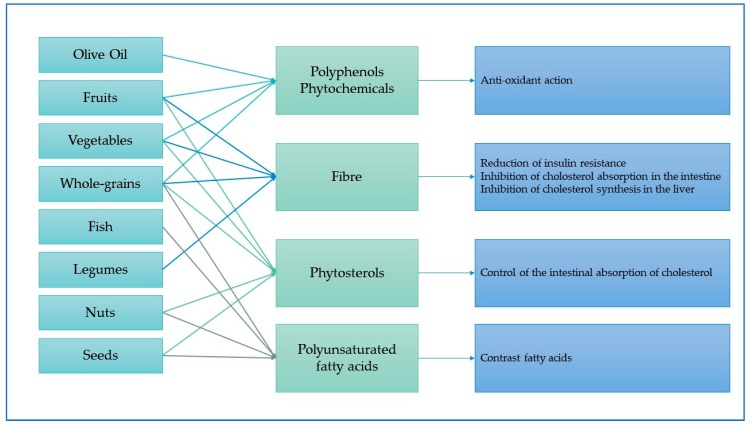
Mechanism between Mediterranean diet components and beneficial effects—Source information: Lăcătușu et al. (2019).

Determining the mechanism of interaction between Mediterranean foods and cancer requires an in-depth study of macro- and micro-elements contained in each food or produced by each of them in response of a certain cooking of method. In Figure 6, we reported main Mediterranean foods, their biochemical components and their consequent action. Overall, polyphenols and phytochemicals, contained in many Mediterranean foods, have a protective and antioxidant effect, even to balance fatty acids contained in some food of the Mediterranean diet, as olive oil. Fiber contained in whole grains, vegetables, legumes, and fruit, as well as increasing antioxidant vitamins and phytochemicals, reduce insulin resistance, inhibit cholesterol absorption in the intestine and cholesterol synthesis in the liver [24,88].

Specifically, we noted that many Mediterranean foods contribute to reducing cancer risk with a series of mechanisms that reduce tumor cell growth (i.e., fish intake), anti-oxidative and anti-inflammatory effects (i.e., fruit, vegetables, and olive oil), increase chemoprotective effects (i.e., olive oil), and inhibit tumor development (i.e., dairy products) [89,90,91] (see Appendix A). Controversial results are emerged about red wine consumption, although recent researches demonstrated as wine micronutrients, such as polyphenols (resveratrol and quercetin, in particular) [83,84] could have positive effects against cancer. Observing a wholeness Mediterranean diet without regarding a single food and pathways leading to a favorable effect on various disease, cancer included, it emerged that this dietary pattern produces the following positive effects [92]:

- lowering of lipid and modulating of their effects;
- anti-inflammatory, anti-oxidative and anti-aggregating effects;
- modulation of cancer-prone mediators (hormones or growth factors);
- reduction, through the changes in amino acid content, of stimulation of hormones or other extra- and intracellular transmitting pathways involved in cancer;
- changes in gut microbiota thanks to a positive and modified production of bacterial metabolites.

## 4. Impact of Mediterranean Diet on Cancer

### 4.1. Breast Cancer

As reported in the introduction, breast cancer is the neoplasia more widespread among women (less than 1% of men suffer from this cancer) so that in 2018 it affected 25% of women with a new diagnosis [2]. Breast cancer risk doubles every ten years until menopause, and the period after that the risk continues to increase but more slowly, though it remains high [93,94].

Environmental factors (e.g., pollution) and lifestyle affect sensibly the breast cancer onset: It was demonstrated a worrying increase of incidence among women in developing countries, historically less subjected to this neoplasia since they are less exposure to environmental and hormonal factors. It seems that this observed increase is due to the “Westernization” of their lifestyle which modifies their habits (e.g., use of alcohol or smoking) and their traditional diets favoring non-healthy diet patterns rich in saturated fats [95].

Mediterranean diets may be a protective factor in the decrease of breast cancer incidence thank to a regular intake of fiber, antioxidants, flavonoids included, vitamins and carotenoids, able to reduce estrogens and to increase level of sex-hormones and therefore to neutralize free radicals, to protect from DNA damages, and to reduce oxidative stress [96].

Several studies underlined the presence of an inverse relationship between high MD adherence and breast cancer incidence. Overall, it emerged that the incidence decreased of 6% in case of high MD adherence measured with arMED (adapted relative Mediterranean diet excluding alcohol) (HR_arMEDhigh_ vs. _arMEDlow_ = 0.94, 95% CI 0.88–1.00), and values of this decrease were slightly higher (7%) for post-menopause women (HR_arMEDhigh_ vs. _arMEDlow_ = 0.93, 95% CI: 0.87–0.99) and significantly higher (20%) in case of BC ER- (ER negative)/PR- (PR negative) HR_arMEDhigh_ vs. _arMEDlow_ = 0.80, 95% CI: 0.65–0.99) [97].

Modifying the determination method of MD adherence, i.e., considering aMED (Mediterranean diet score excluding alcohol) or MDS (Mediterranean diet score), as described by Trichopoulou [53], it emerged that incidence risk considerably decreased (40%) for post-menopause women (HR_MD high_ vs. _MD low_ = 0.60, 95% CI: 0.39–0.93) [94], and achieved 14% or 18% considering a medium or a high level of MD adherence and keeping a moderate alcohol intake (OR_MDS=4-5 vs. MDS=0-3_ = 0.86, 95% CI: 0.76–0.98 and OR_MDS=6-9 vs. MDS=0-3_ = 0.82, 95% CI: 0.71–0.95) [96]. See Table 1 for reviewed studies.

Though many studies had demonstrated that there was a significant relationship between alcohol and breast cancer [98,99], a recent work showed that excluding completely alcohol from the diet pattern could contribute to increase benefits in case of medium adherence to MD (i.e., risk passes from 14% to 19%) (OR_MDS=4-5 vs. MDS=0-3_ = 0.81, 95% CI: 0.71–0.91), but it did not increase or reduce positive effects of MD in case of a high adherence to it (OR_MDS=6-9 vs. MDS=0-3_ = 0.81, 95% CI: 0.70–0.95) [96]. Vice versa, observing the impact of diet on breast cancer using a different diet pattern, it was noted that risk was three-times higher in case of no-healthy diets [100] and it approximately increased of seven-times for those who had preferred fried meat and had avoided stews or other dietic cooking methods [101].

### 4.2. Colorectal Cancer (CRC)

Diet in general and especially Mediterranean diet play a crucial role for colorectal cancer since healthy or non-healthy diet patterns are considered among the most important risk factors in the onset of this neoplasia [15,102]. In fact, a better quality of diet, whichever it is as long as healthy, is associated with a low CRC risk [14] independently from hereditary, traditional (e.g., belonging to a specific ethnic group) [103] or environmental factors. Important changes in the lifestyle (e.g., introduction of a regular physical activity and a healthy diet) and the exclusion of smoking could determine a reduction in CRC incidence of 70% [14].

It emerged that fiber, calcium and a regular intake of garlic may represent protective elements reducing CRC risk [2,17,104] while no-healthy diet patterns, e.g., those with a high intake of meat or alcohol, represent negative elements increasing CRC incidence [2,16,105,106,107].

A higher adherence to MD was able to reduce of about 30% and 45% CRC risk in men (OR_Q4_ vs. _Q1_ = 0.71, 95% CI: 0.55–0.92) and in women (OR_Q4_ vs. _Q1_ = 0.65, 95% CI: 0.40–0.77) respectively [108], and the percentage of risk significantly decreased also examining tumor sides (proximal colon OR_Q4_ vs. _Q1_ = 0.70, 95% CI: 0.51–0.97, distal colon OR_Q4_ vs. _Q1_ = 0.65, 95% CI: 0.48–0.89, and rectum OR_Q4_ vs. _Q1_ = 0.60, 95% CI: 0.45–0.81) [108].

On the contrary, a Western diet patterns, i.e., a “westernized” alimentary regimen characterized by a high consumption of dairy, meat, refined cereals, sweets, energy drink, and sauces [35,108], increased CRC risk for men (OR_Q4_ vs. _Q1_ = 1.45, 95% CI: 1.11–1.91) and women (OR_Q4_ vs. _Q1_ = 2.02, 95% CI: 1.44–2.84) respectively, and had a significant and negative impact in case of rectal cancer (OR_Q4_ vs. _Q1_ = 1.46, 95% CI: 1.05–2.01).

To understand how much a diet could impact on the CRC onset, it was noted that the lowest adherence to Western diet could reduce of 1/3 distal colon cancer and of 1/4 rectal cancer [108], since a low intake of sugared drink and read meat could decrease the risk to develop advanced polyps risk (OR_sweety drinks_ = 0.56, 95% CI: 0.36–0.87; OR_red meat_ = 0.63, 95% CI: 0.42–0.95) [13].

Vice versa, a high MD adherence could reduce of 1/5 and 1/4 distal colon and rectum cancer cases respectively [108], allowing a significant reduction of advanced polyps (OR_MDS = 3-4_ = 0.34 (0.17–0.65), OR_MDS = 5-7_ = 0.22 (0.11–0.43); OR_MDS = 8-10_ = 0.18 (0.07–0.47)) [13] and a decrease of CRC risk of 11% (OR = 0.89, 95% CI: 0.86–0.91) for each 1-point increase of MD score [109].

Using different adherence scores, as MMDS (Modified Mediterranean Diet Score), CSMMDS (Centre-Specific MMDS), and Italian Mediterranean Index, did not affect the predictive capacity of MD in the evaluation of the risk: An increase in the MD score, calculated with one of the just mentioned scores, could reduce CRC risk of 11%, 8% and 50% respectively even if confounding variables were considered [15,111].

The high protective capacity of MD was confirmed also in a recent Ratjen’s study (2017) in which they demonstrated as a high MD adherence (measured with MMDS) could reduce mortality rate in CRC patients (HR_highest quartile_ vs. _lowest quartile_ = 0.48, 95% CI: 0.32–0.74) and, if sex or age variables were considered, it could be possible observing a reduction of mortality rate of 11% and 12% respectively for each 1-point increase of MMDS (HR_highest quartile_ vs. _lowest quartile_ = 0.88, 95%CI: 0.81–0.96) [110]. See Table 2 for reviewed studies.

### 4.3. Prostate Cancer (PCa)

In accordance with Wilson and Giovannucci’s study (2012), lifestyle and diet pattern are the leading factors in the prevention of the most lethal prostate cancer cases [112]. For this kind of neoplasia, fats, especially animals derived fats and oils, dairy and calcium have a negative effect increasing the PCa incidence [18,19,20,113], while a high intake of fiber and vegetables, soya, legumes, green tea, and tomatoes, in other words an increase of folates, vitamins, especially vitamin C, and of nutrients as lycopene, have a protective effect on prostate, so reducing the cancer risk.

Studies conducted by Kenfield and Richard respectively, confirmed the presence of a strong relationship between diet pattern and prostate cancer risk. Both demonstrated as PCa was less widespread in Mediterranean area than in the Northern Europe ones [115,116]: A high MD adherence not only was inversely associated with a low incidence of prostate cancer, but also it was associated with lower cancer malignancy (44%, OR_high score_ vs. _low score_ = 0.66, 95% CI: 0.46–0.95) [114] and mortality rate for PCa (22%, HR = 0.78, 95% CI: 0.67–0.90) in patients without metastasis [115]. Overall, a high MD score was associated with a low likelihood of PCa (OR = 0.86, 95% CI: 0.77–0.96); thereby, PCa risk decreased until 78% in subjects with the highest MD scores, registering in particular a decrease of 14% for each one-point increase of MD score [85]. See Table 3 for reviewed studies.

### 4.4. Gastric Cancer (GC)

With reference to stomach cancer, although the predominant risk factor is the presence of *Helicobacter pylori* (responsible for 89% of GC cases) [117] and there is an inverse causality problem between risk factors related to diet and gastric cancer (causality determined by disease preclinical symptoms which modify alimentary habits) [118], in this case also it is not possible to exclude a predominant role of diet in the gastric cancer onset [119,120,121].

Observing more closely MD products and connecting them with GC, we may note as Mediterranean characteristics, which can contribute, as protective factors, to reduce GC risk, are mainly represented by a high intake of antioxidants, contained in fresh fruit and vegetables. In fact, antioxidants reduce oxidative DNA damages eliminating free radicals and taking part in many biological changes linked with all cancers (e.g., bioactivation of carcinogens, cell signaling, cell regulation circle, angiogenesis and inflammation), but in this specific neoplasia, a high intake of fresh fruit and vegetable may reduce the negative effect of *Helicobacter pylori* and its consequent damages [72].

Although there are many confounding factors in the onset of the GC cancer (as the just mentioned *Helicobacter pylori*), it was demonstrated as the adherence to a healthy lifestyle, in which the diet represents one of the fundamental elements, reduced significantly GC risk until 51% (HR 0.49, 95% CI 0.35, 0.70), with a higher incidence in case of cardia GC (HR 0.23, 95% CI 0.08, 0.68) than in case of non-cardia GC (HR 0.53, 95% CI 0.32, 0.87), so that 19% of patients would have avoided cancer onset in case of an improvement of their lifestyle (no smoking, moderate alcohol, healthy diet and normal BMI) [122]. A higher adherence to Mediterranean diet could reduce significantly GC incidence: Comparing subjects classified in the lowest category of MD adherence (0–3) with those in the following two categories (medium: 4–5 and high: ≥6) the percentage of risk decreased until 22% and 43% respectively, showing so a significant and inverse trend between MD and GC risk [69].

### 4.5. Bladder Cancer

549.393 new cases of bladder cancer have been diagnosed in 2018 with a global incidence of 3.2% and a stronger predominance among men (4.9%) than among women (1.5%) [2]. For this kind of cancer, diet plays an important role since many metabolites and pollutants (as arsenic contained in drinking tap water) of ingested foods are excreted through the urinary tract and therefore, they come into direct contact with the bladder mucosa [123,124]. Olive oil has a protective role in the reduction of risk of 38% and 50%, respectively, in case of a moderate (OR_Q2_ vs. _Q1_ = 0.62, 95% CI: 0.39–0.99) or a high (OR _Q3_ vs. _Q1_ = 0.47, 95% CI: 0.28–0.78) consumption of this typical Mediterranean product [125].

Analyzing globally the impact of MD on bladder cancer incidence, and also considering some confounding variables (such as smoking, obesity, sex, and age), they demonstrated that there was an inverse relationship between MD and bladder cancer risk with a reduction until 28% in case of a moderate adherence (OR_MDS = 4-5_ vs. _MDS = 0-3_ = 0.72, 95% CI: 0.54–0.98) and until 34% in case of a high adherence (OR_MDS = 6-9_ vs. _MDS = 0-3_ = 0.66, 95% CI: 0.47–0.93), i.e., they could observe a decrease of risk of 11% for each one-point increase in MD score [82].

Examining the effect of confounding factors, it emerged an influence of smoking habits on the impact of MD. This influence was slight and no-significant for non-heavy smokers (bladder cancer risk increases of 15% when it was compared with that reported by non-smokers), but it increased significantly for heavy smokers (more than 20 cigarettes/day). Combining the results, it emerged that the Mediterranean diet impact was stronger for non-smoker patients and when tumor invasiveness was classified as pT1-pT4 [82].

This means that MD benefits and its protective role are more evident if they are considered in a healthy lifestyle context aiming to avoid all risk factors which not only participate in the bladder cancer onset, but also, they could reduce positive effects of a healthy diet. To understand better how much a diet may reduce bladder cancer risk, two studies proved that greater adherence to the Mediterranean diet would have been able to avoid the onset of the disease in 12.6% [82] and 4% [126], respectively, of patients registered subsequently with bladder cancer.

### 4.6. Malignant Tumors of the Female Reproductive System

Ovarian cancer, vulva cancer, vagina cancer, cervical cancer and endometrial cancer are a series of tumors which affect the female reproductive system. While ovarian, vulva, and vagina cancer have a low incidence (1.7%, 0.3%, and 0.1% respectively in 2018) and their risk factors are related with elements not-linked with diet (as anticipated menarche, nulliparity, menopause after 55 years, smoking, and heredity), the etiology of cervical cancer and endometrial cancer could have a relevant correlation with the diet. More specifically, the onset of cervical cancer (the fourth most common cancer as well as the fourth cause of death in the world [127]), is strong correlated with the diet pattern: A regular consumption of fruit, vegetable and therefore of nutrients, as vitamins A, E, C, folates, carotenoids and minerals, may contribute to a reduction of cervical cancer risk [3] thanks to a protective role played by these nutrients to inhibit proliferation of cancerogenic cells and to prevent DNA damages [128,129].

Given the strong relationship between hrHPV (high-risk human papilloma virus) infection and cervical cancer [130], it was demonstrated that MD played a crucial role producing an indirect protective effect in the onset of these neoplasia. In fact, since there is a direct relationship between the Mediterranean diet and the slowdown of progression of hrHPV infection, the Mediterranean diet acts on the infection which, if it is reduced, does not contribute to increase cervical cancer risk. Therefore, a high adherence to MD was able to reduce of 60% the cervical cancer risk (adjOR_MDShigh_ vs. _MDSlow_ = 0.40, 95% CI: 0.21–0.75), although this effect was mediate by a reduction of hrHPV. The same positive benefit was not observable in case of Western diet: In this case a greater adherence represented a risk factor in the onset of cervical cancer (adjOR_Q3_ vs. _Q1_ = 1.77, 95% CI = 1.04–3.54 and adjOR_Q4_ vs. _Q1_ = 1.97, 95%CI = 1.14–4.18) [3].

In regards to endometrial cancer (the fourth most common cancer in European women [131]), 382,069 new cases were observed in 2018 (5.3% of all women affected by cancer). The major factor in the onset of this cancer is identified in an unbalanced and/or prolonged exposure to endogenous or exogenous estrogens (situations happening in case of advanced menopause, nulliparity and in presence of polycystic ovary) [132], since the increase of estrogens, also consequent to some specific therapies, if it is not opposed by progesterone, may increase the mitotic activity of endometrial cells so producing unwanted DNA replications with a consequence rise of probability of mutation [133]. Given the strong correlation between being overweight or obesity and hormonal problems [134], it is possible to suppose an active role of the diet in the prevention on the endometrial cancer onset modifying estrogens production [135]. A regular consumption of vegetable and fruit had a protective effect in onset of endometrial cancer with a reduction of risk of 66% (adjOR_5th quartile_ vs. _1st quartile_: 0.34, 95% CI 0.17–0.68) and of 45% (adjOR_5th quartile_ vs. _1st quartile_: 0.55, 95% CI 0.28–1.06). A high adherence to MD could contribute to reduce risk of 43% (adjOR = 0.57, 95% CI: 0.39–0.86) and of 49% (adjOR = 0.51, 95% CI: 0.28–0.92) in case of moderate and high adherence respectively [4]. Analyzing incidence results considering confounding factors and MD score without reference to levels, it was also demonstrated as an increase of one-point in MD score could produce a decrease of risk of 16% and that this decrease was stronger in case of elderly women or in women no-taking of contraceptives or hormonal therapies [136].

### 4.7. Head-Neck Cancer (HNC)

Incidence of head-neck cancers—oral cavity tumors, oropharynx cancer, hypopharynx cancer, and pharynx cancer—registered a global incidence of 5.2% in 2018. The survival rate at five-years is 40–50% [137]. Although major risk factors are identified in smoking, excessive use of alcohol and in some infections, such as papillomavirus [138], diet plays a crucial role so that some epidemiological studies underlined as a protective effect can be determined by a high intake of fruit and vegetable, especially in case of oral cavity cancer [2,139]. For this reason, a high adherence to MD was inversely associated with a reduction of HNC risk of 20% in men (HR = 0.80; 95% CI: 0.64–1.01) and of 58% in women (HR = 0.42; 95% CI: 0.24–0.74) [138]. This evidence was stronger for oral cavity cancer or pharynx cancer for which the percentage of risk reduction achieved 80% in case of a high level of MD adherence (OR_MDShigh_ vs. _MDSlow_ = 0.20, 95% CI: 0.14–0.21), and became more evident for the young and ex-smokers [76].

In case of nasopharyngeal cancer, it was demonstrated as diet not only played a crucial role [140], but also it showed as a moderate adherence to MD score could reduce risk by 17% (OR_MDS=5_ vs. _MDS≤4_ = 0.83, 95% CI: 0.54–1.25) and a high adherence (MDS ≥ 6) could be able to reduce risk by 34% (OR_MDS≥6_ vs. _MDS≤4_ = 0.66, 95% CI: 0.44–0.99)), thus showing that an increase in adherence to the Mediterranean diet with a score above or equal to six would have been able to prevent 22% of the observed cases [64].

### 4.8. Biliary Tract Cancer (BTC), Pancreatic Tumors

Carcinoma of gall bladder and bile ducts are some of the biliary tract tumors. The first is one of the most malignant cancers because of the lateness of diagnosis, often in advanced phases, and the poor survival rate [141]. For these reasons, it is complex to determine incidence, although an intake of fresh fruit and vegetable together with a low consumption of prepacked foods rich in sodium, may represent a determinant factor in the onset of the disease. In fact, oxidative and inflammatory processes or other carcinogens effects could be decrease thank to an improvement of diet quality which surely influences the appearance of gallstones and reduces obesity, both considered risk factors in the onset of this kinds of cancers [142]. An increase of MD adherence, measured with mMED, was inversely associated with a reduction of risk of extra-hepatic BTC ((OR = 0.41 (0.25–0.67)) and gall bladder cancer (OR = 0.42 (0.23–0.79)) [142].

With attention to pancreatic cancer, considered the seventh worldwide cause of death [143], although it was registered as an higher adherence to MD was a protective factor against cancer onset (OR_MDS__≥6_ vs. _MDS=0-3_ = 0.57, 95% CI: 0.34–0.95, OR_MDS__≥6_ vs. _MDS=0-3_ = 0.51, 95% CI: 0.29–0.92; OR_MDS__≥6vsMDS=0-3_ = 0.48, 95% CI: 0.35–067 values related to three different Italian centers) [75], we noted that there were not many significant correlations between healthy alimentary habits and the onset of pancreatic cancer. For this reason, some studies have been more concentrated to demonstrate how erroneous lifestyles and diet patterns (e.g., smoking, excessive alcohol assumption and low adherence to MD) increased the pancreatic cancer risk. In fact, there was a positive association between low intake of fruit and vegetable and high consumption of red meat and pancreatic risk [144]. At the same time, an excessive consumption of alcohol increased pancreatic risk until 60% and a low adherence to MD could determine a risk rise of 11.9% [145].

### 4.9. Lung Cancer

Lung cancer is the most leading cause of death in the westernized countries and its worldwide incidence is the greatest among the whole cancers (2.09 million of new cases in 2018). Given the strong relationship between smoking and lung cancer and between air pollution and lung cancer, it could seem that this kind of tumor is the least correlated with diet. Although there is a high complexity to identify a significant relationship between diet pattern and lung cancer incidence [5,100,146,147], two studies demonstrated that some healthy diet patterns (Healthy Eating Index-2010 (HEI-2010), Alternative Healthy Eating Index-2010 (AHEI-2010), alternate Mediterranean diet score (aMED), dietary approaches to stop hypertension (DASH)) were inversely associated with lung cancer (HEI-2010: HR = 0.83 (0.77–0.89), AHEI-2010: HR = 0.86 (0.80–0.92), aMED: HR = 0.85 (0.79–0.91), DASH: HR = 0.84 (0.78–0.90), MDS: HR_7-9_ vs. _0-3_ = 0.64 (0.45–0.90)) [5,148]. Beneficial effects of MD were evident in case of heavy smokers: The MD score was inversely correlated with lung cancer in smokers, so that an increase of MD level adherence (medium or high) could determine a reduction of risk of either 62% (HR_7-9_ vs. _0-3_ = 0.38 (0.19–0.75)) or 90% (HR_aMED≥8_ vs. _aMED≤1_ = 0.10 (0.01–0.77)) [148,149].

## 5. Conclusions

Examining jointly a series of significant studies, related to last 10 years, about the association between the Mediterranean diet and cancer risk or its incidence, it emerged that diet may represent a determinant factor in the cancer onset, especially when MD adherence is not an occasional or moderate diet pattern, but it is a regular and constant lifestyle. In our analysis of MD benefits, it emerged as diet is a protective factor against the cancer onset, especially when there is a high intake of olive oil, fresh fruit and vegetables, thanks to antioxidant and anti-inflammatory properties of these foods. Therefore, it is necessary to increase prospective studies in which it is evaluated how adherence to MD reduce cancer risk taking in account a series of exogenous variables as geographical areas (therefore their pollution), lifestyle, hereditary factors and origin of foods.

The lack of information about the quality of consumed alimentary products seems represent a limitation of research; if on the one hand the adherence to the MD is characterized by a high intake of some foods, the lack of information about the quality of the same foods (e.g., product treated or not-treated with chemical agents, antibiotics or hormones) could modify results reducing the benefits of MD; this means that the lack of significance in some studies could not be determined by an actual lack of relationship, but caused by the presence of noxious substances, which, in turn, are risk factors in the cancer onset, if not even the main reason for cell mutation. If this biological traceability of products could appear difficult in the case of fruit and vegetables, it becomes particularly complex for meat and, practically impossible, for fish (especially for sea fish). Although the Mediterranean diet recommends a low intake of meat, so that noxious products could have a low impact, it is necessary to underline as the total amount of hormones and antibiotics, ingested by fruit, vegetable, and meat, has an enormous noxious impact on human health (e.g., estradiol is class A1 carcinogens, progesterone, if ingested, causes damages to immune system until immunodepression, as well as it is class 2B carcinogens, androgen is class 2A carcinogens, antibiotics increases antibiotics resistance and altered intestinal flora raising cancer risk, especially for some kind of neoplasia as colorectal cancer) [150,151,152].

Many efforts have been made to reduce the use of hormones and antibiotics in agriculture and livestock farming (e.g., the promotion of organic farming (Reg. (CE) n° 834/2007), prohibition of hormones and antibiotics for auxin purpose in animals (96/22/EC, art. 11 del Reg CE n.1831/2003)), but it is necessary to evaluate in future studies the levels of adherence to the Mediterranean diet together with the origin of foods and their possible level of pollution.

In conclusion, from this narrative review it emerged clearly that the Mediterranean diet may contribute to the reduction of cancer onset in the worldwide population since it is characterized by a series of foods that, due to their antioxidant and anti-inflammatory properties, are able to prevent and counteract DNA damages and slow down the development of various forms of cancer, affecting negatively cell proliferation. Following this dietary pattern could be particularly complex both because of the difficult availability of the foods and because of the objective difficulty of satisfying the world demand for Mediterranean products ensuring, at the same time, the quantity and quality of the offered products. It is, therefore, necessary to invite governments and institutions to evaluate the cost-benefits of promoting education programs for proper nutrition in order to reduce cancer onset through prevention campaigns that educate a healthy lifestyle in which diet is one of the strengths, remembering that “The Mediterranean diet is a set of traditional practices, knowledge and skills passed on from generation to generation and providing a sense of belonging and continuity to the concerned communities” [29].

## Figures and Tables

**Table 1:** Summary of studies reviewed and relationships with statistical significance between MD score and breast cancer.

Study	Study Characteristics	MD Adherence	Objective	Statistical Method	Results
Buckland [97] (2013)	Prospective Study Case-control Sample: 335,062 women Period: 1992 to 2000 Place: Europe	arMED	Incidence of cancer	Cox proportional hazard regression model	HR_arMEDhigh_ vs. _arMEDlow_ = 0.94 (0.88–1.00) HR_arMEDhigh_ vs. _arMEDlow_ = 0.93 (0.87–0.99) HR_arMEDhigh_ vs. _arMEDlow_ = 0.80 (0.65–0.99)
Van den Brandt [94] (2017)	Prospective Study Case-control Sample: 62,573 women aged 55–69 years Period: 1986–2007 Place: Netherlands	Mediterranean Diet Score	Incidence of cancer	Cox proportional hazard regression model	HR_MD high_ vs. _MD low_ = 0.60, 95% CI: 0.39–0.93
Turati [96] (2018)	Prospective Study Case-control Sample: 6426 women Period: 1991–2008 Place: Italy and Switz	Mediterranean Diet Score	Incidence of cancer	Logistic regression	OR_MDS=4-5 vs. MDS=0-3_ = 0.86 (0.76–0.98) OR_MDS=6-9 vs. MDS=0-3_ = 0.82 (0.71–0.95) OR_MDS=4-5 vs. MDS=0-3_ = 0.81, (0.71–0.91)

**Table 2:** Summary of studies reviewed and relationships with statistical significance between MD score and colorectal cancer.

Study	Study Characteristics	MD Adherence	Objective	Statistical Method	Results
Castello [108] (2018)	Multicase-control study Sample: 5138 Period: 2008–2013 Place: 11 Spanish provinces	A posteriori score	Incidence of cancer	Logistic regression	Men: OR_Q4_ vs. _Q1_ = 0.71 (0.55–0.92) Women: OR_Q4_ vs. _Q1_ = 0.65, (0.40–0.77) Proximal colon: OR_Q4_ vs. _Q1_ = 0.70 (0.51–0.97) Distal colon: OR_Q4_ vs. _Q1_ = 0.65 (0.48–0.89) Rectum: OR_Q4_ vs. _Q1_ = 0.60, (0.45–0.81)
Fliss-Isakov [13] (2018)	Case-control study Sample: 783 patients Period: 2010–2015 Place: Israel	A posteriori score	Incidence of cancer	Multivariate logistic regression	OR_MDS = 3-4_ = 0.34 (0.17–0.65), OR_MDS = 5-7_ = 0.22 (0.11–0.43); OR_MDS = 8-10_ = 0.18 (0.07–0.47)
Rosato [109] (2016)	Case-control study Sample: 10,549 patients Period: 1985–1991 Place: Milan (Italy)	Mediterranean Diet Score	Incidence of cancer	Unconditional logistic regression	OR = 0.89, 95% CI: 0.86–0.91 (for each 1-point increase of MD)
Ratjen [110] (2017)	Prospective cohort study Sample: 1404 CRC patients Period: 2004–2007 Place: Northern Germany	A posteriori score	Mortality rate in CRC patients	Cox proportional hazard regression model	HR_highest quartile_ vs. _lowest quartile_ = 0.48 (0.32–0.74) HR_highest quartile_ vs. _lowest quartile_ = 0.88 (0.81–0.96) (for each 1-point increase of MD)

**Table 3:** Summary of studies reviewed and relationships with statistical significance between MD score and prostate cancer.

Study	Study Characteristics	MD Adherence Measurement	Objective	Statistical Method	Results
Schneider [114] (2019)	Prospective study Sample: 2258 patients Period:2004–2009 Place: North Caroline, Louisiana (USA)	Mediterranean Diet Score	Incidence of cancer	Multivariate logistic regression	OR_high score_ vs. _low score_ = 0.66 (0.46–0.95)
Kenfield [115] (2015)	Prospective study Sample: 47,867 men Period: 1986–2010 Place: USA	Mediterranean Diet Score	Mortality rate in patients without metastasis	Cox proportional hazard regression model	HR = 0.78 (0.67–0.90)
Russo [85] (2018)	Case-control Sample: 356 patients Period: 2015–2016 Place: Catania (Italy)	MEDILITE score	Incidence of cancer	Multivariate logistic regression	OR = 0.86 (0.77–0.96) (for each 1-point increase of MD score)

## Appendix A

**Table A1:** Elements linked with Mediterranean Food, effect of elements on cancer and cancer risk for each element.

Typical Foods	Elements	Function	Cancer
**Fruits & Vegetables**	Antioxidants and micronutrients (carotenoids, vitamin C, vitamin E, selenium, dietary fiber, dithiolthiones, glucosinates, polyphenols, protease inhibitors, allium compounds, plant sterols, and limonene)	Anti-tumorigenic effect	Less risk of: -Epithelial cancer -Digestive tract cancer -Breast cancer -Female genital tract cancer -Urinary tract cancer
**Fish**	Long-chain omega-3 fatty acids docosahexaenoic acid and eicosapentaenoic acid	Reducing tumor cell growth Modulation of transcription factor activity and signal transduction Alteration of oestrogen metabolism	Less risk of: -Liver cancer -Colorectal cancer
	Heterocyclic amines and polycyclic aromatic hydrocarbons may be formed when fish is cooked on a grill or barbecue	Production of mutagenic chemicals	High risk of stomach cancer
**Olive oil**	Polyphenols (oleuropein and hydroxytyrosol)	Antioxidant activity, anti-inflammatory and anti-mutagenic effects	Less risk of: -breast cancer -ovarian cancer -upper aero-digestive tract cancer -colorectal cancer
	Oleic acid, poly unsaturated fatty acids (PUFA), low n-6 PUFA/n-3 PUFA ratio	Chemoprotective effect	
**Meat**	Heterocyclic amines and polycyclic aromatic hydrocarbons formed when meat is cooked at high temperatures	Carcinogens	High risk of: -colorectal cancer -nasopharynx cancer -ung cancer -pancreatic cancer -bladder cancer -esophagus cancer (squamous cell carcinoma) -stomach (no-cardia) cancer
	Haem iron, present in high level	Promotion of tumorigenesis by stimulating the endogenous formation of carcinogenic N-nitroso compounds	
	High-temperature cooking of red and processed meats may enhance production of advanced glycation endproducts (AGEs).	Produce several cancer-promoting effects	High risk of pancreatic cancer
	Consumption of meat may lead to insulin resistance and hyperinsulinemia, promoting growth of cancer cells	Promoting growth of cancer cells	
**Whole grains**	Provide various nutrients: vitamin E, selenium, copper, zinc and bioactive non-nutrient compounds (lignans, phytoestrogens, and phenolic compounds), and dietary fiber	Anti-carcinogenic properties, as anti-oxidative activity Reduce insulin resistance	Less risk of: -colorectum cancer -upper aero-digestive tract -stomach cancer -breast cancer -ovarian cancer -kidney cancer
	Aflatoxin (mycotoxin produced by molds of the Aspergillus species)	High mutation load in TP3	High risk of liver cancer
**Dairy Products**	Calcium, lactic acid-producing bacteria, vitamin D, linoleic acids, lactoferrin,	Inhibit tumor development	Less risk of: -breast cancer (pre-menopausal and post-menopausal women) -colorectal cancer
	High level of calcium	Downregulating the formation of the biologically active form of vitamin D → increasing cellular proliferation	Higher risk of prostate cancer
**Red Wine**	Phytoalexin presents in grape skin	Antioxidant and cancer chemo preventive agent → inhibiting tumor initiation, promotion and progression	Controversial results about impact
	Resveratrol and quercetin	Modulating cell cycle-regulating proteins Inducing apoptosis in multiple carcinoma cell lines Anti-inflammatory, growth → inhibiting activity and immunomodulation properties	

Information source: World Cancer Research Fund and Grosso et al. (2013).
